# Engineering autoimmune disease models using organoids: Harnessing microenvironmental engineering for precision medicine and immunological recapitulation

**DOI:** 10.1002/btm2.70162

**Published:** 2026-07-30

**Authors:** Chang‐Jin Lee, Yeojin Kim, Misu Kim, Yeri Alice Rim, Ji Hyeon Ju

**Affiliations:** ^1^ Department of Medical Sciences Graduate School of The Catholic University of Korea Seoul Republic of Korea; ^2^ CiSTEM Laboratory, Catholic iPSC Research Center, Seoul St. Mary's Hospital, College of Medicine The Catholic University of Korea Seoul Republic of Korea; ^3^ YiPSCELL Inc. Seoul Seocho‐gu Republic of Korea; ^4^ Division of Rheumatology, Department of Internal Medicine Seoul St. Mary's Hospital, Institute of Medical Science, College of Medicine, The Catholic University of Korea Seoul Republic of Korea

**Keywords:** autoimmune disease modeling, autoimmune organoids, iPSC, precision medicine

## Abstract

Autoimmune diseases—including systemic sclerosis (SSc), systemic lupus erythematosus (SLE), and rheumatoid arthritis (RA)—are increasingly understood as programmable microenvironmental states, wherein evolving changes in matrix mechanics, barrier integrity, interferon and cytokine networks, immune‐complex deposition, and stromal–immune reciprocity progressively reshape tissue behavior. These shifting axes generate nonlinear trajectories of fibrosis, vascular injury, and joint destruction that static or reductionist in vitro systems fail to recapitulate. Organoid and organ‐on‐a‐chip platforms now allow controlled reconstruction of these dynamic microenvironments in human‐derived systems. By integrating iPSC‐derived epithelial, endothelial, stromal, and immune lineages with tunable extracellular matrix (ECM) stiffness and viscoelasticity, perfusable microvasculature, and modular innate and adaptive immune components, these systems reproduce key autoimmune phenomena. These include stiffness‐driven fibroblast activation and endothelial‐to‐mesenchymal transition (EndoMT), interferon‐conditioned barrier collapse, immune‐complex‐mediated injury, and cytokine‐dependent stromal invasion. Patient‐specific induced pluripotent stem cell (iPSC), CRISPR‐based editing of risk alleles, and controlled exposure to sera or autoantibody repertoires provide genetic and immunologic personalization, while multi‐omic profiling, spatial imaging, and machine‐learning analytics enable quantitative alignment between organoid states and patient tissues. By translating pathogenic microenvironmental logic into controllable 3D systems, these platforms establish autoimmune organoids as programmable, human‐relevant tools for mechanistic discovery, therapeutic interrogation, and precision modeling—laying the groundwork for next‐generation, potentially animal‐free pipelines and advancing personalized immunology across SSc, SLE, and RA.

**Translational impact statement**

This review highlights how organoid and organ‐on‐a‐chip platforms can reconstruct the dynamic microenvironmental remodeling that drives systemic sclerosis, systemic lupus erythematosus, and rheumatoid arthritis, which is incompletely captured by conventional cell and animal models. By integrating patient‐specific iPSCs, autoantibody or serum exposure, perfusable multicompartment architectures, and multi‐omic readouts, these human‐derived systems provide a more predictive and personalized framework for modeling autoimmune pathology. Such platforms may accelerate biomarker discovery, improve patient‐specific therapeutic testing, support combination therapy optimization, and advance next‐generation, potentially animal‐free translational workflows in precision immunology.

## 1 INTRODUCTION

Autoimmune diseases such as systemic sclerosis (SSc), systemic lupus erythematosus (SLE), and rheumatoid arthritis (RA) are increasingly recognized not merely as failures of immune tolerance but as disorders driven by progressive reprogramming of local tissue microenvironments.^1^ Across affected organs, evolving changes in extracellular matrix (ECM) mechanics, stromal and endothelial plasticity, interferon‐ and cytokine‐amplification loops, metabolic stress, microvascular instability, and barrier dysfunction collectively reshape immune‐cell recruitment, activation, retention, and effector behavior.^2^ These mechanical, biochemical, and spatial perturbations give rise to distinct, spatially organized pathogenic niches—such as fibrotic consolidation in SSc, interferon‐conditioned barrier fragility in SLE, and cytokine‐driven stromal invasion in RA—that unfold as dynamic, tissue‐level programs rather than isolated molecular defects.

Despite decades of insight from cellular systems and animal models, current preclinical platforms capture only fragments of these complex human microenvironmental states. Murine and reductionist in vitro systems do not reproduce the mechanically fixed and epigenetically stabilized fibroblast programs characteristic of SSc; the chronic type I interferon (IFN‐I)‐conditioned epithelial and endothelial injury that defines SLE; nor the metabolically rewired, invasive fibroblast‐like synoviocytes (FLSs) that drive RA pannus formation.^3^, ^4^, ^5^ In addition, human‐specific pathogenic phenomena—including endothelial‐to‐mesenchymal transition (EndoMT), immune‐complex deposition, complement‐mediated podocyte injury, neutrophil extracellular traps (NETs)‐driven vascular destabilization, and synovium–cartilage crosstalk—depend on matrix architecture, stiffness trajectories, spatial cytokine gradients, and evolving autoantibody repertoires that are difficult to recapitulate in vivo.^6^, ^7^ Conventional platforms also fail to resolve the inter‐ and intra‐patient heterogeneity that shapes disease course and therapeutic response, including variability in interferon signatures in SLE, divergent fibroblast mechanosensitivity in SSc, and functionally distinct fibroblast‐like synoviocyte sublineages in RA.^8^, ^9^, ^10^

Recent advances in organoid and microphysiological engineering now provide an opportunity to reconstruct autoimmune pathology within controllable, human‐relevant microenvironments.^11^, ^12^, ^13^ Tissue‐specific organoids derived from pluripotent or primary cells can be embedded in ECM scaffolds with tunable stiffness and viscoelasticity, interfaced with perfusable and shear‐responsive microvasculature, and integrated with modular innate and adaptive immune compartments.^14^, ^15^ Co‐culture strategies further enable incorporation of macrophages, dendritic cells, neutrophils, and T‐ and B‐cell populations—including autologous immune lineages generated from patient‐specific iPSCs—allowing microenvironmental cues such as cytokine gradients, oxygen tension, mechanical forces, antigenic inputs, and autoantibody repertoires to be precisely controlled.^16^, ^17^, ^18^ Complementary analytical frameworks including single‐cell and spatial transcriptomics, matrisome proteomics, super‐resolution imaging, and quantitative biomechanical profiling enable high‐resolution tracking of engineered microenvironmental states and alignment of engineered tissues with patient‐derived signatures.^19^, ^20^

Still, despite these advances, the field lacks a unified conceptual and engineering framework that explicitly links autoimmune pathogenic logic to reproducible and quantitatively validated model design. Existing organoid and organ‐on‐a‐chip studies often capture individual components—such as fibrosis, interferon signaling, immune‐complex‐mediated injury, or stromal invasion—without integrating the shared and disease‐specific microenvironmental architectures that coordinate disease progression across tissues. This fragmentation limits their ability to reconstruct dynamic disease trajectories, resolve patient‐specific endotypes, and generate predictive insights into therapeutic response.^21^, ^22^

In this review, we therefore frame autoimmune diseases as programmable microenvironmental states: Section 2 outlines the conserved and disease‐specific pathogenic axes of SSc, SLE, and RA. Section 3 translates these axes into engineering principles for organoid and microphysiological systems capable of reconstructing dynamic fibrosis, interferon‐mediated barrier injury, immune‐complex deposition, and stromal invasion. Section 4 integrates ECM design, mechanobiology, immune–stromal spatial patterning, perfusion architectures, and multi‐omic analytical tools into a unified microenvironmental engineering framework. Section 5 discusses validation strategies, remaining biological and technical limitations, and key directions for transforming autoimmune organoids into predictive, personalized, and ultimately animal‐free preclinical systems.

By explicitly linking autoimmune pathogenesis with microenvironmental engineering principles, this review aims to provide a conceptual and practical roadmap for constructing next‐generation autoimmune organoids and organ‐on‐a‐chip systems that capture disease complexity, heterogeneity, and temporal evolution while enabling mechanistic dissection and translational application.

## 2 AUTOIMMUNE MICROENVIRONMENT LOGIC AND PATHOGENIC AXES

Autoimmune diseases—despite their clinical heterogeneity—are organized by a shared microenvironmental logic in which mechanical forces, cytokine and interferon circuits, nucleic acid sensing, barrier stability, and immune–stromal reciprocity collectively govern tissue behavior (Figure 1a).^23^

**FIGURE 1:**
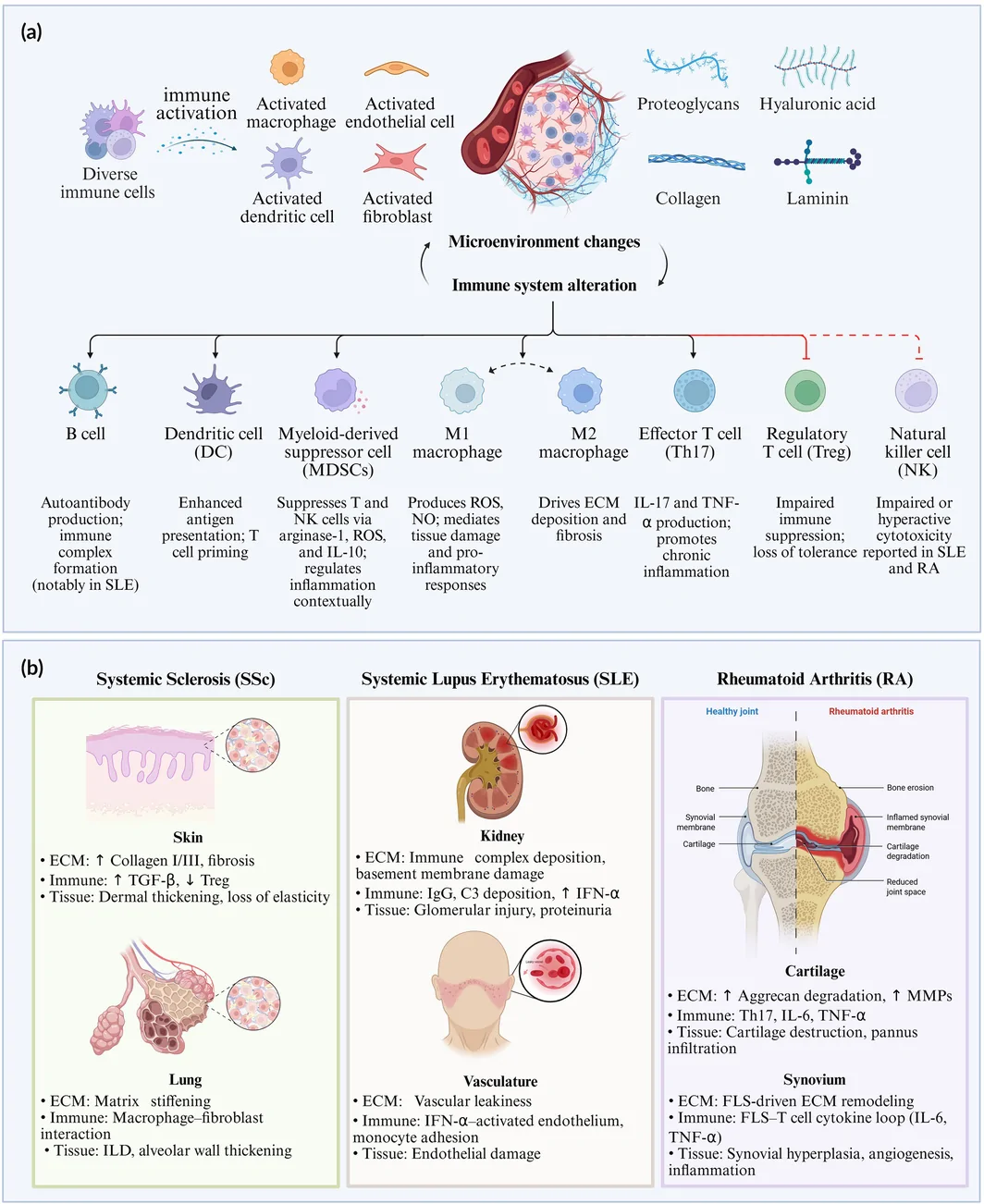
Tissue‐specific microenvironmental remodeling and immunopathology in autoimmune diseases. (a) Core microenvironmental programs that drive autoimmune pathology across tissues. Stromal, endothelial, epithelial, and immune cells undergo coordinated remodeling involving ECM deposition, viscoelastic stiffening, altered barrier integrity, and cytokine/interferon amplification. These processes create spatially organized “inflammatory neighborhoods” that shape immune recruitment, activation thresholds, and effector persistence. (b) Disease‐specific microenvironmental trajectories. Systemic sclerosis (SSc) is characterized by progressive fibroblast fixation, ECM stiffening, and mechanotransductive consolidation in skin and lung, whereas systemic lupus erythematosus (SLE) involves immune‐complex deposition, type I interferon‐driven barrier fragility, and vascular destabilization across kidney, vasculature, gut, and heart. In rheumatoid arthritis (RA), chronic inflammation manifests through cytokine‐saturated synovial niches, invasive FLSs, matrix degradation, and osteochondral destruction. Together, these conserved and disease‐specific axes define the architectural logic that organoid platforms must reconstruct to model autoimmune disease with human fidelity.

Rather than arising from isolated immune defects, pathology emerges within spatially organized inflammatory niches composed of stromal, epithelial, endothelial, and immune cell populations embedded within structured extracellular matrix (ECM) architectures and biochemical gradients.^24^ These niches are sustained by breakdown of immune tolerance and persistent activation of autoreactive T and B lymphocytes, together with antigen‐presenting cells and tissue‐resident stromal cells, forming self‐reinforcing signaling networks that drive fibrosis, interferonopathy, and tissue invasion. This breakdown involves defects in both central and peripheral tolerance checkpoints, allowing self‐reactive lymphocytes to escape deletion and become chronically activated within tissue microenvironments.

Within these environments, autoimmune pathology is mediated through three principal mechanisms: (i) loss of immune tolerance and expansion of autoreactive lymphocytes, (ii) immune‐complex‐mediated and cytotoxic tissue injury, and (iii) chronic cytokine‐driven stromal remodeling. These processes are not independent but are functionally integrated through cellular and molecular signaling networks, forming self‐reinforcing pathogenic circuits that generate disease‐specific microenvironmental states.^25^

This section delineates three conserved microenvironment–immune circuits and describes how their differential weighting gives rise to the pathogenic axes of systemic sclerosis (SSc), systemic lupus erythematosus (SLE), and rheumatoid arthritis (RA) (Figure 1b). Importantly, these axes define the mechanistic basis for the engineering principles discussed in Section 3, linking cellular and molecular pathogenesis to model design requirements.

### 2.1 Shared microenvironment–immune circuits

Across autoimmune diseases, pathology arises from three tightly coupled modules that operate through coordinated cellular and molecular interactions. These modules can be conceptualized as interconnected layers involving immune cell dysregulation, signaling pathway activation, and tissue remodeling processes. Specifically, these modules are driven by defined cellular and molecular mechanisms, including autoreactive lymphocyte activation, cytokine signaling networks, and effector pathways that mediate tissue injury and remodeling.

#### 2.1.1 ECM remodeling and mechanotransduction

Remodeling of ECM composition and architecture—including changes in collagen alignment, crosslinking, stiffness, and viscoelasticity—actively drives cell‐state transitions rather than merely reflecting tissue damage.^26^ Mechanical cues are transduced through integrin clustering and activation of FAK–RhoA–ROCK signaling, leading to cytoskeletal tension and nuclear translocation of YAP/TAZ and MRTF‐A, which in turn remodel chromatin accessibility and activate mechanoresponsive transcriptional programs. This mechanotransductive signaling cascade links extracellular matrix stiffness to intracellular transcriptional reprogramming, enabling fibroblasts to maintain activation independently of transient inflammatory stimuli.

At the cellular level, fibroblasts and myofibroblasts act as central effectors of this axis, acquiring persistent activation states through epigenetic stabilization of profibrotic enhancers. This process establishes “mechanical memory,” whereby fibroblasts maintain ECM production independently of transient inflammatory signals. Concurrent stromal plasticity, including endothelial‐to‐mesenchymal transition (EndoMT), epithelial–mesenchymal transition (EMT), and adipocyte‐to‐fibroblast conversion, further expands matrix‐producing cell populations and reinforces the structural basis of inflammatory niches.^27^

#### 2.1.2 Barrier destabilization

Epithelial and endothelial barriers regulate immune compartmentalization, but in autoimmune disease these interfaces undergo progressive destabilization driven by inflammatory signaling. Type I interferons and cytokines such as TNF‐α and IL‐6 disrupt tight junction proteins and cytoskeletal organization, while mitochondrial dysfunction reduces cellular resilience to mechanical and oxidative stress.^28^ These alterations increase epithelial and endothelial permeability, facilitating immune‐cell infiltration and exposure of subendothelial structures to circulating immune components.

Barrier failure permits immune‐complex deposition, complement activation, and leukocyte infiltration, thereby amplifying local inflammation. Mechanistically, engagement of Fc receptors by immune complexes and complement‐mediated membrane attack further injure barrier‐forming cells, creating a feed‐forward loop of permeability and tissue damage. The resulting increase in paracellular transport reshapes cytokine and chemokine gradients, driving spatial propagation and amplification of inflammatory signaling within tissues.^29^

#### 2.1.3 Self‐reinforcing cytokine, interferon, and metabolic circuits

Persistent activation of innate and adaptive immune pathways establishes self‐sustaining inflammatory circuits. Nucleic acid sensing through TLR7/9 and cGAS–STING pathways drives type I interferon production, which enhances antigen presentation and promotes activation of autoreactive B cells and T cells.^30^ Plasmacytoid dendritic cells (pDCs) play a central role in sustaining type I interferon production, while activated T helper subsets, particularly Th17 cells, further amplify inflammatory signaling.

Cytokine networks—particularly IL‐6–JAK–STAT signaling—support Th17 differentiation and sustain chronic inflammatory responses, while macrophages and dendritic cells produce TNF‐α, IL‐1β, and other mediators that reinforce immune activation. Failure of regulatory mechanisms, including impaired Treg function and dysregulated B‐cell tolerance, further amplifies these circuits. Dysregulation of immune checkpoint pathways, such as CTLA‐4 and PD‐1, further lowers activation thresholds of autoreactive lymphocytes.

Metabolic rewiring, characterized by accumulation of lactate, succinate, and stabilization of HIF1α, integrates with immune signaling to maintain inflammatory states. Stromal cells within these environments adopt trained‐immunity–like epigenetic programs, enabling sustained cytokine production even in the absence of initiating stimuli.^31^, ^32^

Collectively, these modules form an integrated microenvironmental system in which ECM mechanics, immune signaling, and barrier integrity are linked through nonlinear feedback loops. These feedback interactions establish persistent disease states by coupling stromal activation, immune amplification, and barrier dysfunction into self‐reinforcing pathological circuits. Each autoimmune disease selectively amplifies specific components of this system, generating distinct pathogenic axes.

### 2.2 Pathogenic axis of systemic sclerosis: fibrotic and mechanotransductive remodeling

Systemic sclerosis is defined by a fibrotic–mechanotransductive axis in which vascular injury, fibroblast fixation, and ECM stiffening reinforce one another. Early microvascular destabilization—driven by oxidative stress, anti‐endothelial autoantibodies, endothelin‐1, and disturbed shear stress—induces endothelial apoptosis and EndoMT, reducing angiogenic capacity and promoting capillary rarefaction.^33^ TGF‐β signaling acts as a central molecular regulator linking immune activation to fibroblast‐driven extracellular matrix deposition and fibrosis. These vascular alterations are accompanied by immune‐mediated endothelial activation and cytokine signaling that further promote fibroblast activation and matrix deposition. Lymphatic dysfunction increases interstitial pressure and primes perivascular fibroblasts.

Within this compromised niche, fibroblasts undergo metabolic and epigenetic reprogramming marked by increased glycolysis, mitochondrial dysfunction, and persistent YAP/TAZ and MRTF‐A activation.^34^ Activated macrophages and dendritic cells further amplify fibroblast activation through the secretion of profibrotic mediators such as TGF‐β and IL‐6, linking immune activation to stromal remodeling. Mechanotransduction‐related enhancers remain accessible, enabling fibroblasts to sustain collagen production even after soluble cues decline. Progressive ECM accumulation further increases stiffness and anisotropy, amplifying fibroblast activation and consolidating “mechanical memory.” Hypoxia‐driven recruitment of macrophages and pDCs further stabilizes this fibrotic circuit.^35^

SSc axis summary: microvascular injury → fibroblast fixation → ECM stiffening → mechanotransductive memory → fibrotic consolidation.

Engineering implications: SSc models must incorporate dynamically stiffening matrices, EndoMT‐competent vasculature, and oxygen‐/pressure‐responsive microenvironments.

### 2.3 Pathogenic axis of systemic lupus erythematosus: immune‐complex, interferon, and barrier amplification

SLE follows an immune‐complex–interferon–barrier amplification axis that integrates nucleic acid sensing, complement activation, and metabolic stress. Nucleic acid‐containing immune complexes—modulated by valency and charge—deposit along vascular and epithelial interfaces.^36^ These complexes activate pDCs, neutrophils, and stromal cells through Fcγ receptors and TLR7/9, generating robust type I interferon production.^37^ pDCs sustain type I interferon production, which enhances autoreactive B cell activation and reinforces the formation of immune complexes. Autoreactive B cells are the primary source of these immune complexes, linking adaptive immune activation to interferon‐driven innate responses. Spatial transcriptomics reveals persistent interferon “hubs” within barrier networks where interferon‐stimulated genes (ISGs) remain highly accessible and junctional maintenance is impaired.^38^

Interferon‐conditioned epithelial and endothelial layers exhibit reduced mitochondrial reserve and increased sensitivity to mechanical stress, complement attack, and immune‐complex injury. Type I interferon signaling induces ISGs that enhance antigen presentation and further promote B cell activation and autoantibody production, reinforcing the interferon amplification loop. Barrier breakdown accelerates paracellular trafficking of immune complexes, complement proteins, and leukocytes. NET‐associated oxidized mtDNA further activates cGAS–STING, intensifying interferon signaling and vascular dysfunction.^39^

SLE axis summary: immune‐complex deposition → interferon hyperactivation → metabolic‐barrier collapse → systemic propagation.

Engineering implications: SLE platforms require IC‐perfusable vasculature, interferon‐responsive barrier tissues, and complement‐competent microvascular modules.

### 2.4 Pathogenic axis of rheumatoid arthritis: cytokine amplification, stromal reprogramming, and matrix destruction

RA centers on a cytokine‐saturated stromal–invasive axis emerging from interactions among the synovium, cartilage, and bone. Autoantibodies against citrullinated and carbamylated antigens activate macrophages and neutrophils through Fcγ receptors, triggering production of TNF, IL‐1β, IL‐6, and GM‐CSF.^40^ IL‐6–JAK–STAT signaling establishes a central feedback loop that promotes Th17 differentiation and sustains chronic inflammatory responses within the synovium. Concurrent hypoxia and VEGF/HIF1α‐driven angiogenesis expand inflammatory vasculature and enhance leukocyte infiltration.^41^

Within the synovial niche, FLSs undergo metabolic and epigenetic reprogramming‐marked by glycolysis, mitochondrial stress, AP‐1/NF‐κB enhancer activation, and apoptosis resistance‐ and differentiate into invasive subsets.^42^, ^43^ Synovial fibroblasts function as active effector cells by producing pro‐inflammatory cytokines and matrix‐degrading enzymes, thereby directly driving joint destruction. Distinct FLS subpopulations coordinate immune cell recruitment and matrix degradation through chemokine secretion and protease activity, linking stromal reprogramming to immune amplification. Lining‐layer FLS promote cartilage degradation through MMPs and ADAMTS proteases, while sublining FLS recruit immune cells via CXCL12 and GM‐CSF.^44^ These stromal programs activate RANKL‐dependent osteoclastogenesis, driving bone destruction. Altered cartilage viscoelasticity and mechanical loading further accelerate matrix damage.^45^

RA axis summary: autoantibody‐driven innate activation → cytokine saturation → invasive stromal reprogramming → cartilage/bone degradation.

Engineering implications: RA models require heterogeneous FLS subsets, cytokine‐rich niches, degradable cartilage matrices, and osteoclast modules.

## 3 TRANSLATING PATHOGENIC AXES INTO ORGANOID AND ORGAN‐ON‐A‐CHIP MODELS

The pathogenic axes described in Section 2 provide the mechanistic and architectural blueprint for reconstructing autoimmune tissue states in vitro. Rather than being defined by isolated molecular lesions, SSc, SLE, and RA progress through self‐reinforcing microenvironmental trajectories.^16^ These include progressive ECM stiffening and endothelial–mesenchymal transitions in SSc, immune‐complex‐driven interferon amplification and barrier breakdown in SLE, and cytokine‐saturated stromal invasion in RA. Because these trajectories arise from nonlinear interactions among matrix mechanics, immune circuits, metabolic stress, and vascular plasticity, they cannot be accurately modeled using conventional monolayers or static 3D aggregates.^46^

Organoid and organ‐on‐a‐chip platforms therefore must function as adaptive microenvironmental systems. In these systems, mechanical, biochemical, and immune cues evolve along disease‐relevant axes rather than being imposed as static inputs.^46^ In practice, this requires integrating defined cellular components, disease‐relevant cytokine environments, and controllable physical parameters that collectively enable the emergence of pathological microenvironmental states. Ideally, the pathological states characteristic of SSc, SLE, and RA should emerge as system‐level properties of the engineered tissue ecosystem. This section outlines how pathogenic axes translate into engineering specifications, summarizes disease‐aligned microphysiological strategies (Table 1), and discusses personalization approaches that support patient‐relevant autoimmune modeling, while quantitative validation frameworks are addressed in Section 4.

**TABLE 1:** Autoimmune disease–specific in vitro models by target organ.

Disease	Target organ/tissue	Model type	Cellular composition	Key induction signals/cytokines	Modeling strategy	ECM & immune features	Engineered element	Applications	References
Systemic sclerosis (SSc)	Skin	iPSC‐derived 3D skin organoid	iPSC‐derived dermal fibroblasts + keratinocytes (3D layered structure)	TGF‐β, IL‐6, patient serum	Differentiation of patient‐derived iPSCs into fibroblasts and keratinocytes; co‐culture and air‐liquid interface culture to generate fibrotic skin organoids	↑ Collagen I/III, ↑ α‐SMA, TGF‐β1/SMAD and GSK‐3β signaling, fibroblast hyperproliferation	High‐stiffness hydrogel; Air–liquid interface culture	Recapitulation of dermal fibrosis for disease modeling and high‐throughput drug screening (raloxifene as anti‐fibrotic candidate)	^47^
	Lung (alveoli)	hPSC‐derived alveolar organoid	Alveolar epithelial type I & II cells (AEC1, AEC2), progenitors, mesenchymal cells	TGF‐β, CTGF	Stepwise differentiation of hPSCs into alveolar epithelial and mesenchymal cells; TGF‐β1‐induced fibrosis	↑ Collagen I/III, ↑ α‐SMA, ↑ fibronectin; ERK and SMAD signaling activation; fibroblast–epithelial interaction	Gel‐based 3D alveolar scaffold; Tunable stiffness ECM	Fibrotic lung modeling; screening of anti‐fibrotic drug (NP‐011); macrophage‐mediated collagen clearance	^48^
	Vasculature	3D microvessel‐on‐a‐chip (OrganoPlate)	Human microvascular endothelial cells (HMVECs) in collagen‐I ECM with bidirectional perfusion	TGF‐β, TNF‐α, SSc serum	Culture of HMVECs in perfused collagen‐I matrix; stimulation with pro‐fibrotic (TGF‐β) and pro‐inflammatory (TNF‐α) cytokines; exposure to SSc patient serum	EndoMT, vessel loss, ↑ ICAM‐1/VCAM‐1, ↑ TGF‐β, ↑ TNF‐α, serum‐induced anti‐angiogenic effect	Microfluidic perfusion chip; Collagen‐I hydrogel matrix	Modeling microvascular destabilization and rescue screening using TGF‐β/ALK5 and TNF‐α inhibitors; personalized response to patient sera	^49^
Systemic lupus erythematosus (SLE)	Kidney (glomerulus)	iPSC‐derived kidney organoid	Podocytes, mesangial cells, endothelial cells in 3D nephron‐like structures	Immune complexes, IFN‐α, anti‐dsDNA	Differentiation of human iPSCs into nephron‐like structures followed by exposure to SLE serum and anti‐dsDNA antibodies to mimic lupus nephritis	Immune complex deposition (IgG, C3), ↑ ICAM‐1, VCAM‐1, IFN‐α/β signature, podocyte injury	Kidney ECM‐like hydrogel; Self‐organizing nephron structures	Modeling glomerular damage and immune‐complex‐mediated injury in lupus nephritis; testing serum‐induced responses	^50^
	Heart (cardiomyocyte)	iPSC‐derived 2D and 3D cardiomyocyte models	iPSC‐derived cardiomyocytes in 2D and 3D format	SLE serum, anti‐Ro antibodies	Differentiation of iPSCs into CMs using 2D monolayer and 3D spheroid protocols; exposure to active SLE serum and anti‐Ro autoantibody	↑ Fibrosis (COL2A1), hypertrophy (BNP), apoptosis (BAX/Bcl2), disrupted calcium signaling, caspase 3/8↑	Spheroid‐based 3D contractile model; Soft cardiac ECM	Modeling SLE‐associated cardiomyopathy and immune‐mediated myocardial damage; analysis of Ro autoantibody effects	^51^
	Gut (ileum/colon epithelium)	Human ileal and colonic epithelial organoids ± PBMC	Epithelial cells (EPCAM^+^), co‐culture with PBMCs from SLE patients	IFN‐α, lupus serum	IFN‐α or lupus serum stimulation; co‐culture with SLE PBMCs	IFN‐I–induced ISG activation (ISG15, CXCL10), tight junction disruption (ZO‐1↓), epithelial apoptosis, TEER↓	Microfluidic gut chip; Flow‐induced shear stress; Collagen I matrix	Modeling of type I IFN‐driven mucosal injury and microbial translocation; testing of IFNAR blockade or anti‐inflammatory interventions	^52^
Rheumatoid arthritis (RA)	Joint/cartilage	Joint‐on‐a‐chip (3D co‐culture)	RA‐derived FLS spheroids + chondrocytes	TNF, IL‐6, IL‐8, VEGF	Co‐culture of RA‐FLSs and primary chondrocytes in microfluidic chip mimicking synovial–cartilage interface; flow‐perfused	↑ IL‐6, IL‐8, VEGF, MMP‐13; synovial inflammation, ECM degradation, cartilage erosion	Microfluidic synovial–cartilage interface; Stretchable membrane	Modeling reciprocal synovial–cartilage crosstalk, joint remodeling, drug testing	^53^
	Synovium	3D synovial “organoid” (BC scaffold‐based)	RA‐derived FLSs: HUVECs: M1 macrophages = 3:1:1	IL‐6, IL‐8, and VEGF	RA‐FLSs, HUVECs, and THP‐1–derived M1 macrophages co‐cultured on bacterial cellulose (BC) scaffold; self‐organization into spheroid‐like structures	ECM mimicry by BC; ↑ FABP4, PI3K/AKT activation, ↑ IL‐6, IL‐8, VEGF; drug resistance	Bacterial cellulose scaffold; 3D immune–stromal integration	Modeling inflammatory synovial microenvironment, angiogenesis, cytokine signaling, anti‐RA drug screening	^54^
	Synovium	Collagen‐embedded multicellular spheroid	RA‐FLSs + ECs + macrophages	TNF, IL‐6, VEGF, synovial fluid	Spheroid of RA‐FLSs, monocyte‐derived macrophages, ECs; stimulated with VEGF/bFGF or RA synovial fluid	↑ TNF, IL‐6; pannus‐like outgrowth; angiogenesis; ↑ MMP‐3; macrophage‐driven inflammation	Collagen‐I hydrogel; Spheroid invasion matrix	Studying inflammatory interactions, matrix degradation, response to biologics	^55^

Abbreviations: AEC1/2, alveolar epithelial type I/II cells; BC, bacterial cellulose; bFGF, basic fibroblast growth factor; CM, cardiomyocyte; EC, endothelial cell; FLS, fibroblast‐like synoviocyte; HMVEC, human microvascular endothelial cell; HUVEC, human umbilical vein endothelial cell; PBMC, peripheral blood mononuclear cell; TEER, transepithelial/transendothelial electrical resistance.

### 3.1 Design logic: converting pathogenic axes into engineering specifications

The pathogenic axes in Figure 1b outline the multiscale microenvironmental programs that autoimmune organoids must reproduce. Recent insights from single‐cell epigenomics, spatial profiling, and mechanobiology show that SSc, SLE, and RA progress through dynamic, self‐reinforcing trajectories shaped by matrix mechanics, vascular stress, nucleic‐acid sensing, metabolic rewiring, and immune–stromal reciprocity (Figure 2). Engineering strategies must therefore capture the *process* of disease evolution, not merely its terminal phenotypes. This requires incorporating specific cell populations, cytokine environments, and dynamic physical cues that collectively reproduce disease‐relevant signaling interactions.

**FIGURE 2:**
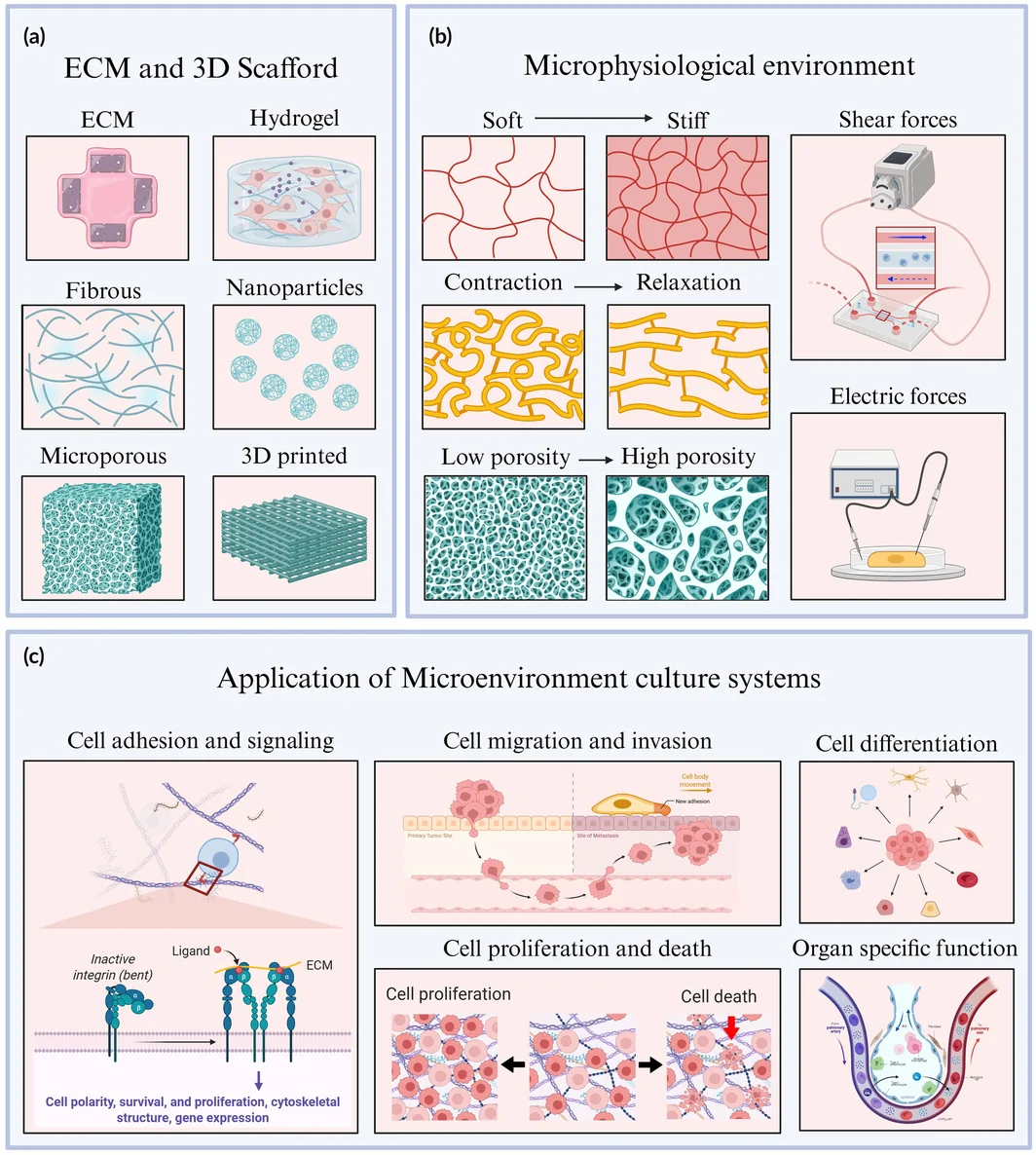
Engineering principles for microenvironmental control in organoid and organ‐on‐a‐chip systems. (a) ECM scaffolds used for organoid engineering, ranging from natural matrices (collagen, matrigel) to synthetic hydrogels and architected biomaterials (fibrous, porous, nanoparticle‐reinforced, or 3D‐printed structures). These materials provide tunable biochemical and mechanical cues that regulate stromal, immune, and barrier cell behavior. (b) Key microphysiological cues that define tissue states—matrix stiffness, viscoelastic relaxation, porosity and architecture, contractility, shear stress, and electrical or mechanical stimulation. Controlled presentation of these cues enables modeling of fibrosis, barrier injury, vascular adaptation, and immune activation. (c) Functional outcomes of engineered microenvironments, including modulation of adhesion, migration, proliferation, survival, lineage specification, invasive behavior, and barrier or vascular function. These engineering modules provide the design vocabulary necessary to reconstruct autoimmune microenvironmental logic in vitro.

In SSc, fibrotic progression is driven by mechanogenomic reprogramming in fibroblasts, including persistent accessibility at YAP/TAZ‐ and MRTF‐A‐linked mechanosensitive enhancers and feedback between mitochondrial reactive oxygen species (ROS), glycolytic bias, and nuclear mechanotransduction. These programs require ECM systems with time‐evolving stiffness, viscoelastic remodeling, and anisotropic collagen architecture. Because microvascular failure precedes fibrosis, endothelial compartments must replicate shear‐sensitive EndoMT, hypoxia responses, oxidative injury, and autoantibody reactivity.^56^ Perfusable architectures enabling controlled hemodynamics, oxygen gradients, and ROS bursts are essential for coupling early vasculopathy to fibroblast activation.^57^

In SLE, pathology emerges from immune‐complex‐driven interferon relay circuits involving FcγR/TLR7/9 activation, cGAS‐STING signaling, and complement amplification. These interactions generate spatially stable interferon “hubs” across epithelial and endothelial barriers. SLE organoids must therefore support flow‐dependent immune‐complex deposition, complement activation, and interferon‐responsive barrier tissues with mitochondrial vulnerability and junctional instability.^58^ Because SLE is a multi‐organ interferonopathy, multicompartment perfusion is required to recreate IFN gradients, cytokine spillover, and immune‐complex trafficking across gut, kidney, heart, and vascular interfaces.^59^

In RA, recent single‐cell analyses reveal stromal lineage diversification, where FLSs adopt invasive, proangiogenic, and osteoclast‐activating phenotypes under chronic TNF, IL‐1β, IL‐6, GM‐CSF, and IL‐17A exposure. These states rely on AP‐1/NF‐κB enhancer activation, glycolytic rewiring, and mitochondrial stress.^60^ To model these trajectories, RA platforms must sustain cytokine‐rich niches, enable synovium–cartilage–bone mechanical coupling, and incorporate degradable cartilage matrices that permit MMP‐ and ADAMTS‐mediated erosion and RANKL‐dependent osteoclastogenesis.^61^

Despite their differences, all autoimmune organoid systems require core capabilities: dynamically tunable ECM mechanics, modular immune integration, perfusable multicompartment architectures, and quantitative readouts that resolve stiffness, barrier integrity, interferon signatures, immune‐complex deposition, metabolic stress, and matrix degradation. These universal modules allow tissues to display emergent microenvironmental behaviors that mirror the nonlinear trajectories characteristic of human autoimmunity, forming the foundation for the disease‐aligned strategies elaborated in Section 3.2.

### 3.2 Disease‐aligned microphysiological strategies

#### 3.2.1 SSc models: dynamic fibrosis and vasculopathy

SSc organoids recapitulate early vasculopathy, fibroblast activation, ECM accumulation, and mechanical consolidation across skin, lung, and vascular systems (Table 1). iPSC‐derived skin organoids containing keratinocytes and fibroblasts reproduce collagen I/III accumulation, α‐SMA induction, and SMAD/ERK activation following TGF‐β, IL‐6, or SSc serum exposure, while preserving donor‐specific fibroblast epigenetic states that reflect individual profibrotic sensitivity.^47^ Dynamically stiffening ECM systems, including photoresponsive or enzyme‐remodeled matrices, extend these models by allowing direct interrogation of mechanical‐memory acquisition.^62^ Lung organoids driven by TGF‐β/CTGF signaling reproduce parenchymal collagen accumulation and epithelial remodeling, while macrophage co‐culture amplifies IL‐6/OSM/TGF‐β circuits that link immune activation to matrix turnover.^48^, ^63^ Microvascular chips further capture shear stress–dependent endothelial activation, barrier dysfunction, and EndoMT under controlled oxygen tension, ROS exposure, and hemodynamic flow.^49^, ^64^ Remaining limitations include incomplete adaptive immune integration and insufficient culture duration for full fibrotic consolidation.

#### 3.2.2 SLE models: immune‐complex, interferon, and barrier fragility

SLE organoids reconstruct the immune‐complex–interferon‐barrier axis across intestinal, renal, vascular, and cardiac systems (Table 1). Intestinal organoids exposed to lupus serum exhibit robust IFN‐I activation, tight‐junction disruption, reduced goblet‐cell differentiation, and metabolic vulnerability, illustrating the combined effects of interferon and low‐level inflammatory cytokines.^52^ Kidney organoids and glomerular chips complement these systems by modeling immune‐complex deposition, complement‐mediated injury, endothelial activation, and podocyte stress.^50^, ^65^ Cardiac spheroids and vascular models further reproduce interferon‐driven tissue injury, autoantibody‐associated damage, and barrier dysfunction.^51^ Multi‐organ microfluidic platforms extend this logic by enabling analysis of systemic interferon propagation, immune‐complex trafficking, and cytokine spillover across organ interfaces, although current systems still face challenges in modeling flare‐remission dynamics and evolving autoantibody repertoires.

#### 3.2.3 RA models: cytokine‐saturated invasion and joint destruction

RA organoid systems model cytokine‐driven stromal invasion using synovial organoids, multicellular spheroids, and joint‐on‐a‐chip platforms (Table 1). Synovial organoids composed of RA‐derived FLS, macrophages, and endothelial cells organize into persistent TNF/IL‐6/GM‐CSF niches that stabilize invasive FLS phenotypes and pathological angiogenesis, revealing disease features not captured in 2D systems.^54^, ^66^ Multicellular spheroids extend this framework by incorporating cytokine cocktails or synovial fluid to induce angiogenesis, macrophage‐dependent inflammatory amplification, and pannus‐like outgrowth.^55^, ^67^ Joint‐on‐a‐chip platforms further spatially couple synovium‐like tissue with cartilage and bone matrices under flow, enabling analysis of MMP/ADAMTS‐mediated cartilage degradation and RANKL‐dependent osteoclastogenesis as integrated mechanisms of joint destruction.^53^, ^68^ Remaining challenges include modeling long‐term inflammatory cycling, physiological mechanical loading, and stable stromal memory.

### 3.3 Genetic, immune, and serum personalization

Personalization strategies—summarized in Figure 3—enable autoimmune organoid platforms to approximate patient‐specific disease trajectories. iPSC‐derived fibroblasts, endothelial cells, epithelial progenitors, and chondrocytes retain donor‐specific transcriptional and epigenetic features, including genetic risk variants and prior immune exposure.^69^

**FIGURE 3:**
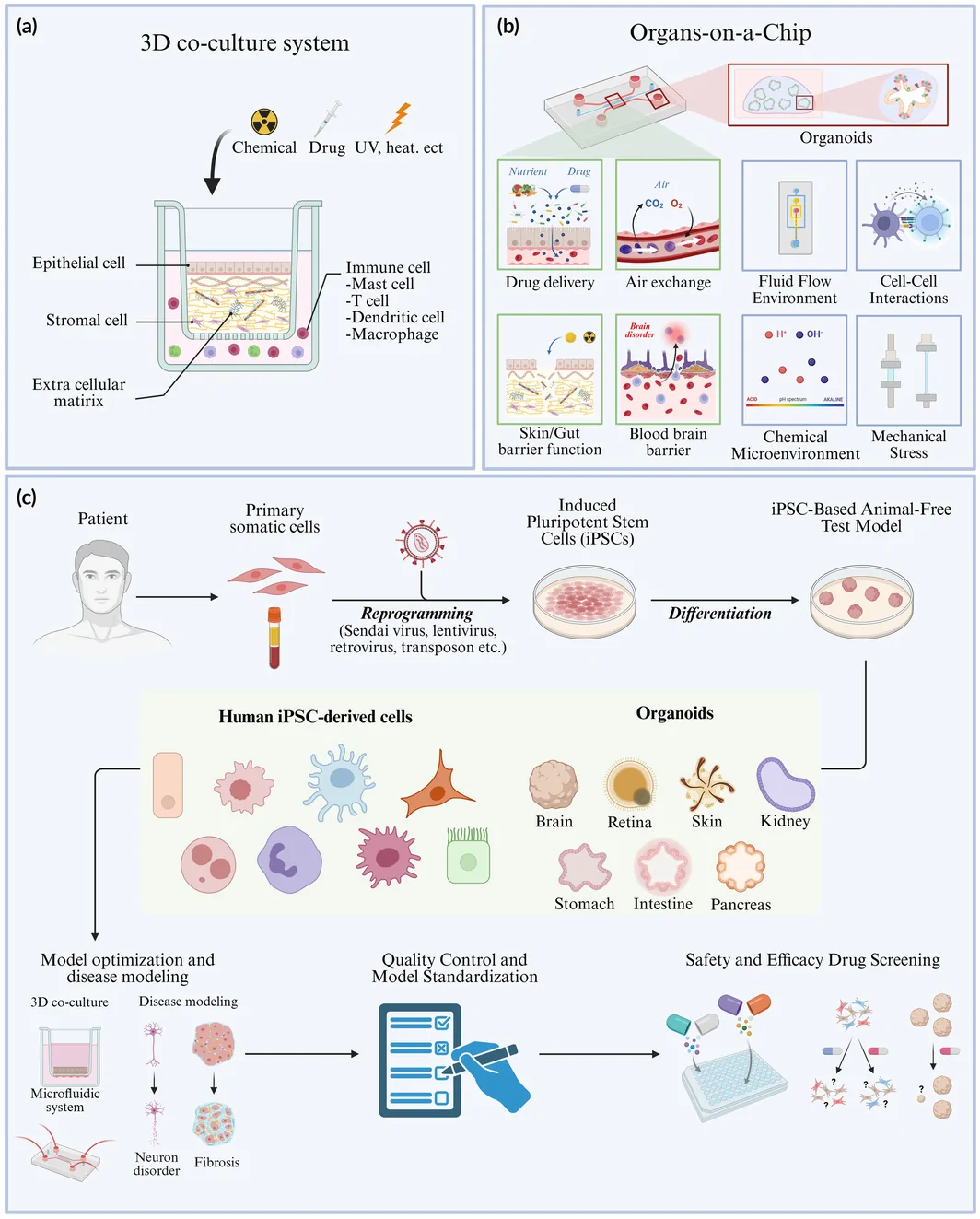
iPSC‐based microenvironmental culture platforms for autoimmune disease modeling and animal‐free testing. (a) Multicellular 3D co‐culture systems integrating epithelial, stromal, endothelial, and immune subsets within ECM‐rich scaffolds. These platforms support controlled exposure to cytokines, autoantibodies, complement, UV stress, or pharmacologic agents, enabling mechanistic dissection of inflammation, fibrosis, and barrier disruption in autoimmune contexts. (b) Organ‐on‐a‐chip technologies that provide perfusable microphysiological environments with regulated nutrient exchange, fluid shear, oxygenation, and mechanical or biochemical stimuli. These interconnected systems allow modeling of vascular injury, immune‐cell trafficking, interferon or cytokine propagation, and cross‐tissue communication. (c) Patient‐specific iPSC pipelines for animal‐free autoimmune disease modeling. Somatic cells are reprogrammed into iPSCs, differentiated into tissue‐specific lineages, assembled into organoids or organ‐on‐a‐chip platforms, and validated using multi‐omic and functional readouts. This workflow enables standardized, high‐content, and personalized modeling of fibrosis, vasculopathy, immune‐complex injury, inflammatory circuits, and therapeutic response.

In SSc, fibroblasts carrying alterations in TGF‐β, PDGFR, or IL11 signaling exhibit heightened profibrotic sensitivity, accelerated myofibroblast differentiation, exaggerated YAP/TAZ activation, and persistent collagen production.

In SLE, epithelial and endothelial cells demonstrate augmented IFN‐I responsiveness, elevated basal ISG expression, and increased barrier fragility.

In RA, stromal cells adopt hyperplastic, cytokine‐responsive profiles resembling pathogenic FLS subsets identified by single‐cell genomics.

CRISPR‐based editing enables direct interrogation of disease genes. Introducing lupus‐risk variants (DNASE1L3, IRF5, STAT4, and TLR7) enhances interferon responsiveness, while correcting pathogenic alleles restores barrier integrity.^70^, ^71^ Editing HLA‐DRB1 shared‐epitope sequences or PTPN22 variants in RA organoids modulates T‐cell help and Th17 polarization.^72^ Manipulating mechanotransduction regulators such as ITGA11 or CAV1 alters contractility and EndoMT susceptibility in SSc fibroblasts.^73^

Integration of patient‐matched immune cells provides another personalization layer. Autologous pDCs, macrophages, Th17 cells, peripheral helper T cells, γδ T cells, and autoantibody‐producing B cells generate individualized immune–stromal dynamics.^74^ Serum‐based personalization adds a third layer: exposure to patient sera enriched for ACPA, anti‐Ro, anti‐RNP, anti‐PDGFR, or high‐IFN signatures produces donor‐specific fibrosis, interferon activation, barrier disruption, or matrix degradation.^52^, ^75^

Multi‐omic characterization links organoid phenotypes to patient tissue states. Single‐cell RNA‐seq, ATAC‐seq, matrisome proteomics, cytokine profiling, and spatial imaging enable direct comparison with biopsy signatures.^76^ Iteratively tuning organoid conditions until transcriptomic and structural profiles converge yields organoid digital twins capable of patient‐specific therapeutic testing.

## 4 ENGINEERING MICROENVIRONMENTS FOR AUTOIMMUNE ORGANOIDS

Reconstructing autoimmune pathophysiology in vitro requires more than assembling lineage‐appropriate cell types; it requires the deliberate engineering of microenvironments capable of behaving as diseased tissues—dynamic, spatially structured, mechanically responsive, and immunologically active.^77^ As outlined in Section 3, the pathogenic axes of systemic sclerosis (SSc), systemic lupus erythematosus (SLE), and rheumatoid arthritis (RA) emerge only when stromal, vascular, epithelial, and immune compartments operate within physical and biochemical contexts that support fibrotic stiffening, interferon‐conditioned barrier fragility, and cytokine‐saturated stromal invasion. In this framework, autoimmune pathology arises not from isolated molecular cues but from the convergence of matrix mechanics, cytokine and chemokine gradients, vascular shear, oxygen tension, tissue topology, and immune–stromal reciprocity.

Whereas Section 3 defined the disease‐relevant functions that autoimmune organoids must reproduce, this section focuses on how those functions can be engineered and quantitatively controlled. Guided by the design modules summarized in Figure 2 and interpreted through the validation framework in Table 2, autoimmune organoids can be viewed as microenvironmental systems whose performance depends on defined mechanical, biochemical, spatial, and analytical parameters. ECMs must undergo quantifiable remodeling and stiffening; epithelial and endothelial barriers must support controlled injury; immune niches must sustain cytokine‐ and autoantibody‐dependent feedback; and multicompartment architectures must propagate mechanical and inflammatory cues under defined flow. To organize this engineering landscape, we outline four domains of microenvironmental design: ECM mechanics and architecture (Section 4.1), immune–stromal integration and spatial patterning (Section 4.2), perfusion and multicompartment organization (Section 4.3), and multi‐omic and computational integration (Section 4.4).

**TABLE 2:** Mechanistically relevant analytical strategies in autoimmune organoid models.

Pathological mechanism	Related disease(s)	Key readouts	Advanced analytical technologies	Clinical significance	References
ECM remodeling and fibrotic stiffening	SSc, RA	↑ Collagen I/III, fibronectin, α‐SMA, porosity index, ACTA2, matrix compaction	AFM, SHG imaging, hydrogel contraction assay, traction force microscopy	Anti‐fibrotic drug screening; prediction of patient‐specific fibrotic trajectory; identification of fibrosis susceptibility phenotypes	^78^
TGF‐β/SMAD pathway activation	SSc	↑ p‐SMAD2/3, CTGF, COL1A1, luciferase activity	Phospho‐flow, luciferase assay, ChIP‐qPCR, SMAD‐reporter lines	Evaluation of TGF‐β‐targeted therapeutics; biomarker discovery for active fibrotic signaling	^79^
Type I interferon‐driven inflammation (ISG Signature)	SLE	↑ ISGs (IFIT1, MX1, ISG15), ↑ STAT1/2 phosphorylation, ↓ TEER, MHC‐II↑	scRNA‐seq, ISG panel, phospho‐array, TEER assay	Prediction of IFN‐blockade responsiveness; stratification of IFN‐high versus IFN‐low patient subsets	^80^
Th17/Treg imbalance and cytokine loops	RA, SLE	↑ IL‐17A, IL‐6, IL‐23, TNF‐α, ↓ FOXP3, ↑ RORγt, ↑ Th17:Treg ratio	Flow cytometry, CyTOF, multiplex ELISA, spatial IF	Assessment of immunosuppressant efficacy; discovery of novel cytokine/immune modulators; patient‐level inflammatory phenotype profiling	^81^
Autoantibody‐driven immune complex deposition	SLE	↑ IgG, C1q, C3 deposition, ↑ ROS, complement activation, podocyte injury	IF staining, lectin staining, TEER, ROS imaging, permeability assay	Testing of anti‐complement/B‐cell–directed therapies; quantification of patient‐specific autoantibody pathogenicity	^82^, ^83^, ^84^, ^85^
Cartilage destruction and ECM segradation	RA	↑ MMP‐13, ADAMTS5, ↓ COL2A1, ↑ COL10A1, aggrecan loss, apoptosis markers	Gel zymography, fluorescent MMP sensor, TUNEL, proteolysis assay	Evaluation of cartilage‐protective or anti‐degradative therapies; modeling progression of erosive joint disease	^86^, ^87^
Endothelial–mesenchymal transition (EndoMT)	SSc	↓ VE‐cadherin, ↑ ZEB1, SNAI1, ICAM‐1/VCAM‐1, α‐SMA↑	Microfluidic flow, confocal imaging, EMT qPCR, transwell adhesion	Predicting vasculopathy severity; evaluating endothelial‐targeting antifibrotic therapies	^88^, ^89^
Barrier integrity loss (epithelial and endothelial)	SSc, SLE	↓ TEER, ↓ ZO‐1/VE‐cadherin, ↑ permeability, ↑ monocyte adhesion	TEER assay, IF staining, FITC‐dextran permeability, flow adhesion	Screening barrier‐restoring agents; assessing mucosal or vascular vulnerability in autoimmune flare cycles	^90^, ^91^
Immune cell infiltration and cell–cell crosstalk	SSc, SLE, and RA	CD3^+^/CD68^+^ infiltration density and spatial distribution, cytokine gradients, CD40L, ICOS, LFA‐1, indices of immune‐mediated tissue injury	3D multiplex IF, spatial mapping, CellPhoneDB ligand–receptor analysis	Profiling immune–stromal interactions; evaluating immunomodulatory therapies; identifying pathogenic immune clusters	^92^, ^93^
Fibroinflammatory signaling via FABP4–PI3K/AKT Axis	RA	↑ FABP4, ↑ p‐AKT (Ser473), ↑ MMP‐3, ↑ IL‐6, ↑ fibroblast migration	Western blot, ELISA, IF, phospho‐array, live‐cell tracking	Identifying invasive FLS phenotypes; testing metabolic or PI3K/AKT‐pathway inhibitors as RA therapeutics	^94^, ^95^, ^96^

Abbreviations: ADAMTS, a disintegrin and metalloproteinase with thrombospondin motifs; AFM, atomic force microscopy; ISG, interferon‐stimulated gene; MMP, matrix metalloproteinase; SHG, second‐harmonic generation; TEER, transepithelial/transendothelial electrical resistance.

### 4.1 Engineering the ECM: stiffness, architecture, and viscoelasticity

The extracellular matrix (ECM) is the primary design variable controlling how stromal, epithelial, endothelial, and immune cells sense and adapt to their environment. Effective ECM systems must therefore function not merely as structural supports but as programmable microenvironmental actuators whose biochemical and mechanical properties can be precisely defined, dynamically tuned, and quantitatively measured.^97^ For autoimmune organoids, this requirement is particularly stringent because SSc, SLE, and RA each exhibit distinct and evolving ECM pathologies that must be recapitulated with engineering precision—ranging from the sub‐kilopascal, interferon‐sensitized fragility of SLE basement membranes to the progressively stiffened, linearly aligned collagen matrices of SSc dermis and the viscoelastic, protease‐remodeling synovium characteristic of RA.

A central engineering requirement is the ability to reproduce these disease‐specific stiffness landscapes using synthetic hydrogels whose crosslinking kinetics, network density, and mechanical behavior are tunable on demand. Synthetic materials such as PEG‐based hydrogels, thiol‐ene or methacrylated matrices, and polyacrylamide/PEG hybrids make it possible to modulate stiffness in a stepwise or continuous manner—an attribute emphasized throughout hydrogel‐engineering literature, where mechanical strength, stress relaxation, and degradation kinetics are identified as major determinants of cell behavior and tissue organization.^98^ These adjustable hydrogels enable fibroblasts, endothelial cells, and immune cells to experience rigidity trajectories analogous to in vivo fibrosis or synovial expansion, revealing how fibroblasts acquire long‐lived mechanical memory, how EndoMT thresholds shift along stiffness gradients, and how RA FLSs adopt invasive phenotypes under chronic mechanical loading.^99^, ^100^ Importantly, the use of synthetic hydrogels allows stiffness to be matched quantitatively to diseased tissue ranges: SSc dermal stiffening often exceeds tens of kilopascals; RA synovium requires viscoelastic dissipation to reproduce cyclic loading and MMP‐mediated remodeling; and SLE barrier fragility is best modeled with sub‐kilopascal basement‐membrane‐like hydrogels.

Beyond stiffness magnitude, viscoelasticity—including stress relaxation, creep, and dissipative mechanical behavior—is a defining feature of autoimmune microenvironments.^101^ Synthetic hydrogels can be engineered to display tailored viscoelastic profiles through chemical, physical, enzymatic, or irradiation‐based crosslinking methods, enabling fine control of relaxation times, reversible bonding, and network plasticity—all properties known to modulate integrin clustering, cytoskeletal tension, nuclear deformation, and lineage‐specific transcriptional reprogramming.^98^ These features are essential for modeling SSc dermis or fibrotic lung stroma, where dissipative mechanics regulate fibroblast activation, and for RA synovium, where viscoelastic ECM supports high migratory and contractile capacities of FLS and immune infiltrates. Likewise, microarchitectural cues such as fiber alignment, pore anisotropy, and nanotopography further shape force propagation and spatial niche organization, enabling engineered ECMs to mimic the collagen linearization of SSc or the directional pannus advance in RA.^102^, ^103^, ^104^

ECM degradability is equally essential. Autoimmune tissues undergo simultaneous matrix deposition and proteolysis; thus ECMs must respond to disease‐relevant proteases—including MMPs, aggrecanases, and cathepsins—rather than remain inert.^105^, ^106^ Synthetic hydrogels incorporating protease‐cleavable linkers, a strategy widely applied in engineered tissue scaffolds, allow real‐time quantification of RA cartilage erosion, SSc matrix turnover, and the transition points at which fibrosis becomes mechanically consolidated.^107^, ^108^, ^109^ Furthermore, engineered ECMs can act as biochemical organizers, with functionalized polymer backbones binding cytokines, chemokines, growth factors, or nucleic acid motifs to generate localized microdomains enriched in TGF‐β, CXCL12, CCL2, or innate‐immune ligands.^110^, ^111^ These domains recapitulate the structured inflammatory niches observed in SSc‐associated fibrotic aggregates, SLE interferon hubs, and the cytokine‐saturated synovial microenvironments of RA.

To ensure that engineered ECMs behave as calibrated disease microenvironments rather than uncontrolled substrates, they must be paired with quantitative mechanical and biochemical characterization tools. second‐harmonic generation (SHG) imaging resolves fibrillar collagen architecture; atomic force microscopy (AFM) and Brillouin microscopy map stiffness and viscoelastic properties; fluorescent protease reporters quantify MMP‐ and cathepsin‐mediated degradation; and matrisome proteomics reveals biochemical remodeling signatures. When interpreted alongside the validation framework summarized in Table 2, these tools transform fibrosis, mechanical signaling, and matrix turnover into tunable, measurable engineering parameters, enabling autoimmune organoids to reproducibly reconstruct the dynamic ECM programs that drive disease progression.

### 4.2 Immune–stromal integration and spatial patterning

Autoimmune pathology is driven not by uniform immune activation but by the spatial organization, density, and persistence of immune–stromal niches. Hallmark examples include perivascular interferon hubs in SLE, fibrotic immune aggregates in SSc, and invasive pannus fronts in RA, where immune cells infiltrate, accumulate, and interact with tissue‐resident stromal compartments to sustain chronic tissue damage.^112^ Engineering autoimmune organoids therefore requires not only inclusion of relevant immune lineages but also precise spatial control over immune localization, activation state, and communication with stromal and parenchymal cells in a manner that is quantitatively definable and experimentally reproducible.^113^

A first design pattern involves innate immune hubs, established by directing macrophages, dendritic cells, and neutrophils into defined regions relative to stromal matrices or vascular interfaces. Micropatterned co‐cultures, photo‐patterned hydrogels, and chemokine‐laden ECMs can position pDCs near fibroblast‐rich matrices to recreate SSc‐like IFN‐α niches, or localize NETosis‐prone neutrophils adjacent to epithelial barriers to model SLE‐associated barrier injury.^114^, ^115^, ^116^ Endothelial‐lined microchannels with tunable ICAM‐1 and VCAM‐1 expression further regulate monocyte adhesion and transmigration under flow.^117^ Within these systems, immune infiltration can be quantitatively assessed by volumetric immune‐cell density, penetration depth from vascular boundaries, and infiltration kinetics, transforming immune recruitment from a qualitative observation into a measurable engineering parameter.

A second pattern focuses on adaptive immune aggregates, including T–B cell zones and Th17‐enriched niches. Microfluidic architectures enable compartmentalized culture of T follicular helper‐B cell clusters that communicate with parenchymal organoids through defined microchannels, permitting spatially regulated autoantibody diffusion, immune‐complex formation, and complement activation.^118^, ^119^ In RA models, Th17 cells embedded within synovial‐like ECMs generate IL‐17A‐ and GM‐CSF‐dominated cytokine microdomains that drive fibroblast‐like synoviocyte activation, invasion, and pannus expansion.^120^, ^121^ These systems allow immune–stromal interactions to be quantified through immune–stromal contact frequency, dwell time within pathogenic niches, and associated cytokine‐gradient amplitudes.

A third pattern integrates biochemical gradients with ECM architecture to localize immune‐mediated tissue injury. Gradient‐generating microfluidics or photo‐patterned hydrogels impose spatial distributions of IL‐6, CXCL12, CCL2, or interferons that interact with aligned fibrillar matrices to define invasive fronts, perivascular cytokine niches, or focal glomerular injury zones.^122^, ^123^ Such spatially structured cues determine where immune complexes preferentially deposit, where endothelial‐to‐mesenchymal transition initiates, and where FLSs begin to erode cartilage.^124^ In these contexts, immune–stromal crosstalk can be linked directly to functional outcomes, including matrix degradation, barrier failure, and localized cell death.

Beyond immune localization, functional immune attrition and tissue injury represent critical determinants of chronic autoimmune pathology.^125^ Spatially resolved readouts of immune activation, exhaustion, and cytotoxicity—such as CD40L, ICOS, and LFA‐1 expression, exhaustion markers including PD‐1 or TOX, and indices of immune‐mediated tissue injury (e.g., caspase activation, TUNEL positivity, or cytotoxic effector expression)—enable discrimination between transient immune infiltration and sustained pathogenic immune niches.^126^

Recent advances in iPSC‐based immune engineering further expand the scope of immune–stromal modeling. iPSC platforms enable generation of immune components with defined reactivity or immune‐evasive properties, providing a foundation for controlled integration of autoreactive or modulatory T‐cell populations into organoid systems.^127^, ^128^ In particular, advances in hypoimmunogenic and immune‐engineered iPSC platforms have enabled the generation of patient‐matched immune elements, supporting their integration into complex tissue and organoid models.^129^ When combined with spatially engineered organoid microenvironments, such approaches open a path toward modeling antigen‐specific immune infiltration, persistence, and tissue injury dynamics that remain inaccessible to conventional animal models.

To translate spatial immune–stromal organization into a validated disease axis, spatial engineering must be paired with quantitative spatial analytics. Multiplex immunofluorescence, imaging mass cytometry, live‐cell tracking, and spatial transcriptomics enable mapping of immune subsets, ligand–receptor interactions, and niche architecture, while computational inference frameworks integrate these data into coherent immune–stromal interaction maps. When aligned with the quantitative metrics summarized in Table 2, these tools transform immune infiltration, crosstalk, and functional tissue injury into tunable, measurable parameters, allowing autoimmune organoids to recapitulate not only immune presence but the spatial logic and pathogenic consequences of immune architecture in SSc, SLE, and RA. Together, these spatial and functional measurements provide a framework for benchmarking engineered immune niches against patient‐derived tissue phenotypes and for validating immune functionality, persistence, and pathogenicity in autoimmune microenvironments.

### 4.3 Perfusion, vascularization, and multicompartment architecture

Fluid flow, vascular signaling, and cross‐tissue communication lie at the core of autoimmune pathophysiology. Shear stress modulates endothelial activation and EndoMT in SSc; circulating immune complexes drive SLE vasculitis and nephritis; and synovial fluid dynamics influence RA invasion and cartilage erosion.^130^, ^131^, ^132^ Capturing these phenomena requires perfusion systems and multicompartment architectures that emulate microvascular flow and systemic connectivity.^133^

Perfused microfluidic platforms enable precise control of hemodynamics and soluble inputs. Endothelial‐lined channels recapitulate physiological or pathological shear stress, while oxygenation modules impose hypoxic or reoxygenation cycles.^134^, ^135^ These systems regulate leukocyte adhesion, immune‐complex deposition, complement activation, and endothelial barrier function—providing a controlled environment for comparing SSc‐like oxidative stress or SLE‐like immune‐complex injury under matched fluidic conditions.^136^, ^137^

Barrier‐on‐chip and vascular–parenchymal interface systems are essential for modeling SLE. Kidney‐on‐chip devices recreate endothelial–podocyte interfaces, allowing immune complexes to deposit under flow and complement to initiate focal injury.^138^, ^139^, ^140^ Gut–vascular chips simulate mucosal–systemic interferon coupling by exposing epithelia to luminal stimuli while permitting cytokines and immune complexes to diffuse into vascular compartments.^141^

For RA, joint‐on‐a‐chip and osteochondral‐on‐chip systems juxtapose synovium‐like tissues with cartilage‐ and bone‐like matrices under flow and cyclic mechanical loading.^142^ These platforms enable real‐time analysis of pannus‐like invasion, MMP/ADAMTS‐driven cartilage degradation, and osteoclast activity.^143^ They illuminate how cytokine‐rich synovial niches reshape cartilage and bone under dynamic mechanical stress.^144^

At the systems level, linked multi‐organ circuits connect gut, kidney, skin, lung, vasculature, or joint compartments through shared or recirculating perfusion loops. These interconnected systems model how interferon signals propagate, how immune complexes generated in one tissue deposit in another, and how systemic therapies produce tissue‐specific effects. Quantitative evaluation of perfusion behavior includes transepithelial/transendothelial electrical resistance (TEER), permeability, adhesion molecules, cytokine profiles, leukocyte trafficking, and imaging of angiogenesis, barrier failure, and tissue invasion‐aligned with the validation domains in Table 2.

### 4.4 Multi‐omic, imaging, and computational integration

Autoimmune organoids achieve their full interpretive power only when paired with analytical frameworks capable of resolving their cellular, mechanical, and spatial complexity. Autoimmune pathology spans multiple scales—from transcriptional states and metabolic stress to ECM remodeling, barrier failure, and immune‐niche organization—necessitating integrated multi‐omic and computational approaches.

Single‐cell RNA‐seq and ATAC‐seq characterize cell states and regulatory programs, revealing SSc fibroblast subsets with heightened mechanotransduction, SLE epithelial and endothelial clusters primed for interferon responses, and RA FLS subsets with invasive phenotypes.^145^, ^146^, ^147^ Proteomic and matrisomic profiling quantify ECM composition, collagen crosslinking, fibronectin remodeling, and protease activity, while secretome and cytokine profiling map inflammatory networks that can be benchmarked against patient samples.^148^, ^149^

Spatial and live imaging preserve tissue architecture and capture dynamic processes. Multiplex immunofluorescence, imaging mass cytometry, and spatial transcriptomics map immune infiltration, cytokine gradients, ISG expression, endothelial activation, stromal remodeling, and immune–stromal communication.^150^ SHG imaging reveals fibrillar collagen patterns in SSc and RA matrices; AFM and Brillouin microscopy map biomechanics; and live reporters detect NETosis, MMP activity, mitochondrial stress, and barrier integrity.^151^, ^152^, ^153^

Computational integration synthesizes these multi‐layered datasets. Deep‐learning models quantify fibrosis architecture, vascular topology, immune clustering, and invasion dynamics.^154^, ^155^ Ligand–receptor inference and spatial statistics reconstruct intercellular communication networks.^156^, ^157^ Multimodal embeddings integrate imaging, transcriptomic, proteomic, secretomic, metabolic, and mechanical features into latent disease‐state coordinates that mirror clinical trajectories.^158^ These methods enable construction of patient‐calibrated organoid digital twins, which can be iteratively tuned to match individual patient profiles and used to evaluate therapeutic strategies both in vitro and in silico.

Together, these engineering and analytical strategies transform autoimmune organoids from descriptive reconstructions into quantitatively interpretable, programmable disease ecosystems. They provide a framework for validating model fidelity, comparing engineering approaches, and advancing autoimmune organoids as predictive and patient‐relevant platforms for mechanistic discovery and therapeutic development.

## 5 CHALLENGES AND FUTURE DIRECTIONS

### 5.1 Biological limits: maturity, memory, and temporal dynamics

Despite considerable advances, current autoimmune organoid systems remain incomplete reconstructions of the microenvironmental programs that drive SSc, SLE and RA. A central biological limitation is cellular maturity. iPSC‐derived stromal, epithelial, endothelial, and immune cells often retain fetal‐like metabolic and epigenetic states, limiting their ability to adopt the adult‐like activation thresholds required to model stabilized disease behavior.^159^, ^160^ Consequently, organoids only partially reproduce the mechanically locked fibroblast states seen in SSc, the interferon‐conditioned epithelial and endothelial fragility characteristic of SLE, or the cytokine‐saturated, metabolically rewired FLSs that drive RA tissue destruction.

A second constraint is the insufficient modeling of durable stromal and immune memory.^161^ The pathogenic axes summarized in Section 2—progressive ECM stiffening and mechanotransductive consolidation in SSc, recurrent interferon oscillations and immune‐complex propagation in SLE, and chronic cytokine amplification with pannus expansion in RA—operate across months to years in vivo. By contrast, most organoid cultures are limited in longevity and cannot maintain ECM architecture, immune‐cell organization, or barrier integrity for extended periods. These systems therefore capture acute disease features but fall short of modeling slow fibrosis progression, chronic synovitis, or flare–remission cycles.^162^

A related challenge concerns temporal patterning. Disease‐relevant dynamics—oscillatory IFN‐I activity, gradual and anisotropic stiffening, fluctuating cytokine environments—rarely emerge spontaneously in vitro and are rarely engineered explicitly.^163^ Progress will require the development of long‐term stable culture environments equipped with dynamic actuation modules, such as ECMs capable of reversible stiffening–relaxation cycles, programmable cytokine and interferon delivery systems that emulate flare rhythms, and biosensors capable of tracking real‐time shifts in matrix remodeling, YAP/TAZ activity, mitochondrial stress, degradomics, and metabolic load.^116^, ^164^

Finally, current systems provide an incomplete representation of autoantibody repertoires. Pathogenic repertoires—anti‐dsDNA, anti‐Ro/SSA, ACPA, anti‐PDGFR—arise from affinity maturation, class‐switch recombination, epitope spreading, and selective survival within germinal center–like ecosystems.^165^ Most models approximate this complexity by exposing tissues to bulk patient serum, which fails to capture repertoire evolution or tissue tropism. Recent tonsil organoids capable of generating maturing, diversified autoantibody repertoires offer a promising direction, and emerging autoimmune organoid platforms—such as the human coeliac disease organoid system that revealed IL‐7‐driven pathological immune activation—demonstrate how disease‐specific immune circuits can be reconstructed in vitro.^17^, ^119^ However, systematic integration of these immune ecosystems into multicompartment autoimmune organoids remains an unmet need.

Another important biological limitation is the absence of neuroendocrine regulation. In vivo, autoimmune diseases are shaped not only by immune and stromal interactions but also by neuroimmune and endocrine inputs that influence vascular tone, barrier function, inflammatory oscillations, and flare susceptibility. Current organoid and organ‐on‐a‐chip systems rarely incorporate neuroendocrine components or hormone‐responsive regulatory circuits, limiting their ability to model stress‐linked exacerbation, endocrine modulation of immunity, or broader whole‐body disease dynamics.

### 5.2 Engineering and multi‐organ integration constraints

Engineering limitations further restrict the ability of organoid and organ‐on‐a‐chip systems to recreate disease‐relevant microenvironments. Reconstructing fibrosis, barrier collapse, immune‐complex injury, or stromal invasion requires dynamic mechanical tunability, including adjustable stiffness, viscoelastic remodeling, and matrix anisotropy.^166^ Likewise, accurate reproduction of inflammatory landscapes requires spatially patterned gradients of cytokines, chemokines, metabolites, and innate ligands.^167^ Yet many current hydrogels and perfusion platforms lose mechanical integrity, biochemical gradients, or immune viability over time, undermining their ability to model long‐lived microenvironmental transitions.^168^

A deeper bottleneck lies in multi‐organ physiological integration. Autoimmune diseases propagate through systemic circulation of cytokines, immune complexes, metabolites, and hemodynamic forces, which couple gut, skin, kidney, vasculature, lung, and joint tissues. Reconstructing gut–vascular–kidney signaling in SLE, synovium–bone marrow cross‐talk in RA, or skin–vascular feedback in SSc requires linked, recirculating microfluidic systems featuring selectively permeable interfaces, modular immune reservoirs, and computational flow controllers capable of approximating human physiological scaling.^169^, ^170^ Current multi‐organ platforms capture only fragments of these interactions and rarely reproduce directional immune‐complex trafficking, interferon propagation, cytokine spillover, or pressure‐dependent endothelial injury.^171^

In particular, the inability to fully reproduce systemic immune circulation remains a critical limitation. In vivo, immune cells, cytokines, immune complexes, and metabolites continuously traffic between tissues through dynamically regulated vascular and lymphatic routes. Most current platforms capture compartmental coupling only partially and do not yet recapitulate sustained immune‐cell recirculation, tissue‐to‐tissue immune relay, or whole‐system inflammatory propagation with physiological fidelity.

These issues intersect with persistent reproducibility and standardization challenges. Autoimmune organoids display substantial donor‐to‐donor heterogeneity in fibroblast activation thresholds, interferon responsiveness, ECM remodeling kinetics, immune recruitment profiles, and therapeutic sensitivity.^172^ Platform variability—arising from differences in hydrogel chemistry, stiffness ranges, cytokine dosing strategies, microfluidic geometries, and immune integration protocols—further complicates comparison. While Table 2 provides candidate readouts, the field still lacks consensus standards for fibrosis indices, interferon‐response metrics, barrier integrity measurements, and invasion or degradomics panels. Harmonized validation frameworks will be essential for achieving cross‐study reproducibility and clinical credibility.

Practical deployment is also constrained by cost and throughput. Many organoid and organ‐on‐a‐chip systems remain labor‐intensive, technically specialized, and expensive to assemble, particularly when they incorporate patient‐derived iPSCs, multicellular immune co‐cultures, perfused microfluidics, and multi‐omic readouts. These features limit scalability for large drug libraries, longitudinal patient testing, and routine translational workflows. Wider adoption will therefore require advances in automation, modularization, and standardized parallelized culture formats that improve throughput without sacrificing biological fidelity.

### 5.3 Toward predictive and personalized autoimmune microphysiology

For autoimmune organoids to transition from descriptive research tools to predictive, clinically relevant microphysiological platforms, advances across biological, engineering, and analytical layers must converge.^173^ Standardization of iPSC lines, ECM materials, perfusion components, and immune integration protocols—combined with automated organoid assembly and AI‐driven quality control—will be necessary to reduce batch effects and improve reproducibility.^174^

A clinically translational organoid platform must also be quantitatively anchored to patient‐level data. Calibration against biopsy transcriptomes and epigenomes, serum cytokine and autoantibody signatures, mechanical properties observed via imaging or elastography, and longitudinal clinical indicators will ensure that in vitro states align with real patient trajectories rather than generic inflammatory responses.^175^

Within this emerging landscape, the concept of the organoid–patient digital twin is particularly powerful. By tuning matrix stiffening trajectories, interferon or cytokine oscillation schedules, immune‐cell composition, mitochondrial state, and autoantibody exposure to recapitulate a patient's individual molecular and histopathologic profile, an organoid can function as a personalized surrogate capable of forecasting flare propensity, tissue vulnerability, and therapeutic responsiveness.^176^ Such individualized models—grounded in the engineering principles outlined in Figure 2 and validated through the quantitative metrics in Table 2—could enable ex vivo evaluation of antifibrotic therapies for SSc, interferon‐ or complement‐targeted strategies for SLE, and cytokine‐ or degradomics‐based interventions for RA.

Beyond this conceptual framework, these platforms could support clinical decision‐making in several concrete ways. First, patient‐derived organoids may enable prospective prediction of treatment response by testing sensitivity or resistance to biologics, small‐molecule inhibitors, complement‐directed agents, or antifibrotic therapies in a patient‐specific microenvironmental context. Second, integrated molecular and functional readouts from these systems could facilitate biomarker discovery by identifying signatures associated with disease progression, flare risk, tissue vulnerability, or therapeutic response. Third, autoimmune organoids may support optimization of combination therapies by evaluating how multiple agents interact within cytokine‐rich, mechanically evolving, and immune‐active tissue environments, thereby helping to distinguish synergistic from antagonistic treatment strategies.

Realizing this vision will require progress across several grand challenges. These include achieving adult‐like maturation of stromal, epithelial, endothelial, and immune niches; engineering long‐lived microenvironmental memory and flare dynamics; reconstructing evolving autoantibody repertoires within physiologic B‐cell ecosystems; building physiologically scaled multi‐organ circuits capable of systemic cytokine and immune‐complex propagation; integrating neuroendocrine and systemic immune regulatory inputs; establishing standardized mechanistic validation frameworks; and integrating multi‐omic, imaging, and computational tools to construct patient‐calibrated autoimmune digital twins.^177^ As immunology, stem cell biology, biomaterials engineering, and data science continue to converge, autoimmune organoids will move from approximations of disease toward quantitatively tuned, patient‐specific microenvironmental ecosystems—laying the foundation for next‐generation precision immunology and targeted therapeutic development.

## 6 CONCLUSION

Autoimmune diseases such as systemic sclerosis, systemic lupus erythematosus, and rheumatoid arthritis arise not only from dysregulated immunity but also from microenvironmental reprogramming that reshapes how structural, vascular, and immune compartments communicate across tissues. Accordingly, organoid and organ‐on‐a‐chip technologies provide a powerful framework for reconstructing autoimmune pathology as dynamic tissue ecosystems rather than as collections of isolated molecular signals. By aligning ECM design, immune–stromal architecture, perfusion, and multi‐omic analytics with disease‐specific pathogenesis, these systems function as programmable replicas of autoimmune circuits rather than static mimics of tissue structure. Across diseases, models that incorporate dynamic matrix stiffening, interferon‐sensitive barriers, or synovium–cartilage–bone interfaces show that complex phenotypes—including progressive fibrosis, ISG‐driven barrier collapse, and pannus‐mediated erosion—can be recapitulated and perturbed in vitro. Incorporating patient‐derived iPSCs, autoantibody repertoires, and individualized immune landscapes further elevates these platforms into personalized microphysiological models capable of mirroring patient‐specific trajectories.

Despite these gains, key challenges remain. Achieving adult‐like maturation in iPSC‐derived lineages, modeling chronicity and flare cycles, stabilizing long‐term immune niches, and implementing physiologic multi‐organ scaling will be essential for capturing the full spectrum of autoimmune behavior. Additional challenges, including incomplete neuroendocrine integration, limited systemic immune circulation, and cost and throughput constraints, must also be addressed for broader translational deployment. Standardized validation frameworks—covering fibrosis indices, interferon signatures, barrier integrity metrics, quantitative immune infiltration and persistence measures, and indices of immune‐mediated tissue injury—will be necessary for ensuring reproducibility across platforms and for anchoring organoid‐based findings to clinical biomarkers. In parallel, advances in spatial omics, high‐content imaging, AI‐based morphometrics, and organoid–patient digital twin analytics hold promise for converting these platforms into predictive systems that anticipate therapeutic responses.

As engineering, stem cell biology, immunology, and computational modeling continue to converge, autoimmune organoids are poised to evolve from mechanistic discovery tools into translational engines for patient‐specific medicine. By allowing microenvironmental programs to be reconstructed, perturbed, and quantified with human fidelity, these systems offer a path toward targeted antifibrotic therapies for SSc, interferon‐modulating interventions for SLE, and cytokine‐ or degradomics‐based strategies for RA. More broadly, they may support patient‐specific treatment response prediction, biomarker discovery, and optimization of combination therapies in clinically relevant settings. Ultimately, engineering autoimmune microenvironments in vitro will not only deepen mechanistic insight into disease but also accelerate the emergence of precision immunology, stratified therapeutics, and animal‐free preclinical testing.

## AUTHOR CONTRIBUTIONS

**Ji Hyeon Ju:** Writing – review and editing; supervision; funding acquisition; project administration. **Chang‐Jin Lee:** Conceptualization; writing – review and editing; writing – original draft; investigation; methodology; project administration; visualization. **Misu Kim:** Writing – review and editing; resources. **Yeri Alice Rim:** Supervision; writing – review and editing; project administration; funding acquisition. **Yeojin Kim:** Investigation; resources.

## FUNDING INFORMATION

This research was supported by a grant from the Korea Health Technology R&D Project through the Korea Health Industry Development Institute (KHIDI), funded by the Ministry of Health & Welfare, Republic of Korea (grant number: HI22C1314); by a grant from the Korea Health Technology R&D Project through KHIDI, funded by the Ministry of Health & Welfare, Republic of Korea (grant number: RS‐2025‐16068032); by the Korea Technology and Information Promotion Agency for SMEs (TIPA), Ministry of SMEs and Startups, through the Tech Investor Program for Scale‐up (TIPS) (Project No. RS‐2023‐00302955); and by the Basic Medical Science Facilitation Program through the Catholic Medical Center of the Catholic University of Korea, funded by the Catholic Education Foundation.

## CONFLICT OF INTEREST STATEMENT

The authors declare no conflicts of interest.

## Data Availability

Data sharing not applicable to this article as no datasets were generated or analyzed during the current study.
